# Contrasting Patterns of Soil Microbial Communities, Physicochemical Properties and Vegetation Characteristics Along Altitude and Soil Depths in an Alpine Ecosystem

**DOI:** 10.3390/biology15181663

**Published:** 2026-09-19

**Authors:** Xinyu Guo, Xue Yang, Xu Wang, Rui Chong, Shicheng Yan, Fujiang Hou, Peiqiang Yu, Zechen Peng, Tao Ran

**Affiliations:** 1 China-Kazakhstan Belt and Road Joint Laboratory on Grassland Ecological Restoration, College of Pastoral Agriculture Science and Technology, Lanzhou University, Lanzhou 730020, China; gxinyu2024@lzu.edu.cn (X.G.);; 2 Animal Husbandry Workstation, Maqu County, Gannan Tibetan Autonomous Prefecture 747300, China; 3 College of Agriculture and Bioresources, The University of Saskatchewan, 51 Campus Drive, Saskatoon, SK S7N 5A8, Canada

**Keywords:** altitude, soil depth, vegetation, microbial community

## Abstract

Altitude and soil depth were associated with vegetation, soil properties, and microbial communities in alpine ecosystems, yet a comprehensive understanding of how these gradients shape soil microbial and physicochemical characteristics on the Qinghai–Tibetan Plateau remains limited. Along altitudinal gradients (low, mid, and high) and soil depths (0–10 cm, 10–20 cm, 20–30 cm), we systematically investigated vegetation traits, soil physicochemical properties, microbial community composition, functional pathways, and co-occurrence networks. Aboveground biomass, community-weighted height, and soil pH declined with altitude. Soil pH and ammonium nitrogen were significantly associated with bacterial community variation in the RDA analyses. Surface and deeper soil layers differed in bacterial taxonomic composition and predicted functional potentials. Co-occurrence network properties also varied across altitude and soil-depth groups. These findings describe spatial associations among vegetation, soil conditions, and bacterial community patterns during the sampling period. This study improves our understanding of how interacting environmental structure soil microbial communities and influence ecosystem functioning in alpine ecosystems.

## Simple Summary

The Qinghai–Tibetan Plateau is covered by vast alpine grasslands that are vital to local livestock farming. This study explores how changes in altitude and soil depth affect plant nutrient content, soil properties, and the tiny organisms living in the soil. Aboveground biomass declined from low to high altitude, and crude protein from 12.8% to 10.1% along the altitude gradient. Soil pH ranged from 7.6 to 6.4 across altitude and soil-depth groups. Ammonium nitrogen content was significantly higher at high altitude in the 20–30 cm layer compared with low- and mid-altitude soils (*p* < 0.05). Microbial community composition, predicted functional potentials, and co-occurrence network properties differed across altitude and soil-depth groups. These findings demonstrate that spatial variations in altitude and soil depth are associated with forage quality, soil conditions, and microbial community patterns in alpine grasslands.

## 1. Introduction

Soil microorganisms are fundamental regulators of terrestrial ecosystem functioning, mediating organic matter decomposition, carbon sequestration, and nutrient cycling processes that determine ecosystem productivity and stability [1]. Through diverse metabolic strategies, microorganisms regulate the transformation and availability of carbon and nitrogen resources, thereby controlling the feedback between soil processes and vegetation dynamics, which are the main driving forces of soil ecosystems [2]. However, microbial communities are not randomly assembled; instead, their composition and functional potential are shaped by environmental filtering, resource availability, and interactions with vegetation. Consequently, understanding how microbial communities respond to environmental gradients is essential for predicting ecosystem responses to global environmental changes.

Altitude gradients provide natural environmental experiments for exploring microbial adaptation because they integrate multiple ecological drivers, including temperature, precipitation, ultraviolet radiation, vegetation productivity, and soil nutrient availability [3]. Along increasing elevations, declining temperature and altered moisture regimes can modify plant productivity, litter input, and soil physicochemical properties, thereby influencing microbial community assembly and ecological functioning. Previous studies have demonstrated that soil pH, soil organic carbon (SOC), and nitrogen availability are major environmental factors regulating microbial diversity and composition across mountain ecosystems [4,5]. However, altitude-associated environmental changes are often accompanied by alterations in vegetation composition and productivity, making it difficult to distinguish the direct effects of climatic filtering from plant-mediated effects on soil microbial communities.

In addition to horizontal environmental gradients, soil depth represents an important vertical ecological gradient that may strongly contribute to microbial habitat conditions. Surface soils receive continuous inputs of plant-derived carbon through litter decomposition and root exudation, providing abundant resources for copiotrophic microorganisms. In contrast, deeper soil layers generally experience reduced carbon availability, lower oxygen diffusion, and stronger nutrient limitation, favoring microorganisms with oligotrophic strategies and specialized metabolic capacities [6]. Although vertical differentiation of microbial communities has been reported in forest and peatland ecosystems [7,8], most studies have examined either elevational variation or soil depth effects independently.

Vegetation represents another critical component linking environmental gradients with belowground microbial processes [9]. Changes in altitude can alter plant community composition, biomass production, and nutrient inputs, which subsequently influence microbial habitats and biogeochemical processes. For example, alpine meadow vegetation on the Qinghai–Tibetan Plateau (QTP) exhibits strong spatial differentiation along environmental gradients, with variations in dominant species, aboveground biomass (AGB), and plant nutrient characteristics [9,10]. Conversely, microbial-mediated nutrient transformations can regulate plant productivity and vegetation characteristics, forming complex plant–soil–microbe feedbacks [11,12]. However, the contribution of vegetation variation to microbial community assembly across combined altitude and soil depth gradients remains poorly understood.

The QTP represents one of the most environmentally sensitive terrestrial ecosystems, where alpine ecosystems are strongly influenced by climatic gradients and soil heterogeneity. Although previous studies have investigated altitude-associated changes in vegetation, soil properties, and microbial communities across the QTP, the mechanisms underlying the coupled regulation of microbial communities by elevational environmental filtering, vertical soil heterogeneity, and vegetation variation remain unclear. Based on this framework, we hypothesized that (1) bacterial community composition differs among altitudes and soil depths, with altitude-related differences depending on soil depth; (2) variation in bacterial community composition is associated with soil pH, SOC, mineral nitrogen availability, and vegetation characteristics, including AGB and forage nutrient content; and (3) surface soils are relatively enriched in bacterial taxa associated with copiotrophic strategies, whereas deeper soils are relatively enriched in taxa associated with oligotrophic strategies. To evaluate these hypotheses, we selected three alpine regions at low, middle, and high altitudes with similar vegetation types, climatic zones, and soil categories to minimize non-altitude-related variability. We collected forage samples and soil samples from three depths (0–10, 10–20, and 20–30 cm). Our objectives were to examine the effects of altitude and soil depth on soil physicochemical properties and bacterial community composition and identify soil and vegetation variables associated with bacterial community variation.

## 2. Materials and Methods

### 2.1. Study Area and Sample Collection

The study was conducted in August 2023 in Gannan Tibetan Autonomous Prefecture, located on the northeastern margin of the Qinghai–Tibetan Plateau (QTP), Gansu Province, China. This region represents a typical alpine ecosystem characterized by strong environmental gradients associated with altitude changes. Three altitude zones were selected: low (3000–3100 m), middle (3300–3400 m), and high altitude (3500–3600 m). At each altitude, vegetation characteristics were surveyed in 12 spatially separated 1 m × 1 m quadrats (n = 12 per altitude; 36 quadrats in total). Nine of these quadrats at each altitude were also used for soil sampling. At each soil sampling quadrat, soil cores were collected from five positions using a 5 cm diameter soil corer and separated into three soil depths (0–10, 10–20, and 20–30 cm) [13]. The five cores collected from the same quadrat and depth were thoroughly homogenized to form one composite soil sample. We collected 81 composite soil samples in total (3 altitudes × 9 sampling points × 3 soil depths). Each composite sample was divided into two portions: one was used for bacterial 16S rRNA gene sequencing, and the other was used to determine soil physicochemical properties.

The dominant species at low altitude were *Elymus nutans* Griseb, *Kobresia pygmaea* (C.B.Clarke) C.B.Clarke and *Medicago falcata* L., the dominant species at mid-altitude were *Kobresia pygmaea*, *Elymus nutans*, and the dominant species at high altitude were *Potentilla anserina* L., *Kobresia pygmaea*, *Elymus nutans* and *Anaphalis lactea* Maxim. Plant species identity, richness, density, and species-specific height were recorded in each vegetation quadrat. Within each quadrat, vegetation from a 0.5 m × 0.5 m area was clipped at ground level using hand shears and placed in paper envelopes for aboveground biomass (AGB) determination. Sample groups were designated according to altitude and soil depth. L1, L2, and L3 represent soil depths of 0–10 cm, 10–20 cm, and 20–30 cm in low altitudes; M1, M2, and M3 represent soil depths of 0–10 cm, 10–20 cm, and 20–30 cm in middle altitudes; and H1, H2, and H3 represent soil depths of 0–10 cm, 10–20 cm, and 20–30 cm in high altitudes.

### 2.2. Determination of Plant Nutrient Contents

The grass samples were dried for 48 h at 65 °C, weighed using a digital balance, and the AGB (g·m^−2^) was calculated by dividing the dry weight of the grass sample with the sampling area (0.25 m^2^). Thereafter, the dried grass samples were ground through a 1 mm screen for chemical composition determination. The dry matter, crude ash, neutral detergent fiber, acid detergent fiber and ether extract were determined according to the official methods of the Association of Official Analytical Chemists (AOAC International) [14]. Total nitrogen was analyzed using flash combustion and thermal conductivity detection (Model 1500, Carlo Erba Instruments, Milan, Italy), and crude protein content was calculated as N × 6.25 crude protein [14].

### 2.3. Measurement of Soil Physicochemical Properties

Soil physicochemical properties, including pH, electric conductivity, SOC, soil NH_4_^+^-N and nitrate (NO_3_^−^-N) were determined. For pH and electric conductivity measurement, 1 g of each soil sample was extracted with 5 mL distilled water, and the pH and electric conductivity of soil samples were measured in the supernatant using a Hach pH meter (Hach Company, Loveland, CO, USA) and an electrical conductivity (EC) meter (DDSJ-319L, Shanghai, China), respectively. The SOC was determined by the oil bath K_2_CrO_7_ titration method [15]. For NH_4_^+^-N and NO_3_^−^-N determination, fresh soil samples were extracted with KCl and then measured on an UV spectrophotometer (UV-2450, Shimadzu Corporation, Kyoto, Japan) according to the method of Ren et al. [16].

### 2.4. 16S rRNA Gene Sequencing and Data Analysis

Total genomic DNA from samples was extracted using the CTAB (Cetyltrimethylammonium Bromide) method [17]. The concentration and purity of the extracted DNA were monitored using a NanoDrop spectrophotometer (ND-2000; NanoDrop Technologies Inc., Wilmington, DE, USA) and 1% agarose gel, and then the concentration of DNA of each sample was diluted to 10 ng/µL using sterile water. The V3-V4 regions of 16S rRNA genes were amplified with specific primers 341F (5′-CCTAYGGGRBGCASCAG-3′) and 806R (5′-GGACTACNNGGGTATCTAAT-3′). All the PCR reactions were performed as described in the literature [18]. PCR products were mixed in equimolar proportions and then purified with a Gel Extraction Kit (Qiagen, Hilden, Germany). Sequencing libraries were generated using NEB Next^®^ Ultra DNA Library Prep Kit for Illumina (New England Biolabs, Ipswich, MA, USA), with index codes added following the manufacturer’s recommendations. Library quality was assessed on an Agilent 5400 Bioanalyzer (Agilent Technologies, Santa Clara, CA, USA). Thereafter, the library was sequenced on an Illumina NovaSeq platform (Illumina, San Diego, CA, USA) at Wekemo Tech Group (Shenzhen, China) and 250 bp paired-end reads were generated.

Raw paired-end reads were imported into QIIME 2 (version 2022.2) and processed using its DADA2 workflow (DADA2 version 1.22.0) [19,20]. Forward and reverse reads were trimmed by 23 and 26 bp, respectively, and truncated at 249 bp. Reads with expected errors >2 was filtered. Denoising was performed independently for each sample, using 1,000,000 reads to train the error model. Paired-end reads were merged with a minimum overlap of 12 bp, and chimeras were removed using the consensus method. The workflow generated an amplicon sequence variants (ASVs) abundance table and representative sequences. Taxonomy was assigned using the QIIME 2 q2-feature-classifier plugin and the SILVA 16S rRNA reference database (release 138.2) [21,22]. Sequences assigned to mitochondria and chloroplasts were removed before downstream analyses.

The ASVs count table was rarefied to 50,966 sequences per sample, the minimum sequencing depth after processing; all 81 samples were retained. Chao1 richness, Shannon diversity, and Simpson diversity were calculated from the rarefied ASVs table. Beta-diversity was assessed using Bray–Curtis, weighted UniFrac, and unweighted UniFrac distance matrices, followed by principal coordinate analysis and nonmetric multidimensional scaling [23,24]. Functional Annotation of Prokaryotic Taxa (FAPROTAX, v1.2.6) was used to infer predicted functional potentials from ASV taxonomic assignments and abundances [25]. Functional results are described as predicted functional potentials inferred from 16S rRNA gene data rather than directly measured microbial activities.

### 2.5. Co-Occurrence Network Analysis

Microbial co-occurrence networks were constructed separately for each altitude × soil depth combination, including low-, mid-, and high-altitude soils at 0–10, 10–20, and 20–30 cm, resulting in nine networks in R (v4.3). Networks were constructed at the ASV level. To reduce the influence of rare taxa, ASVs with a cumulative relative abundance >0.05% across the samples within each altitude × soil depth group were retained. Pairwise Spearman’s rank correlations were calculated among retained ASVs, and *p* values were adjusted for multiple comparisons using the false discovery rate (FDR) procedure [26]. Associations were defined as significant at |r| > 0.85 and adjusted *p* < 0.05. An undirected co-occurrence network was then constructed using the igraph package (v2.2.1) in R. Network topology was characterized by calculating the number of nodes and edges, average degree, clustering coefficient, mean path length, and modularity, which together describe the structural properties of microbial networks. Network visualization was performed using Gephi (v0.10.1), with edges colored by correlation type and nodes colored by phylum-level taxonomy.

### 2.6. Statistical Analysis

The CWH was calculated on the basis of mean plant height and the percent aboveground net primary production of each plant species in the community according to Quan et al. [27]. On the basis of the mean plant height and the percentage of abundance in the community, the CWH was calculated as:

CWH = iSPi × Hi

where Pi is the percent abundance of community i, Hi is the mean plant height of community i, and S is the number of communities [28].

Prior to statistical analyses, the normality of data distribution was assessed using the Shapiro–Wilk test, and homogeneity of variances was evaluated using Levene’s test. Considering the hierarchical structure of the sampling design, each sampling site was considered as the independent experimental unit. One-way ANOVA, followed by Tukey’s honestly significant difference (HSD) test, was used to compare differences in soil physicochemical properties, plant species richness, density, above-ground biomass (AGB), and community-weighted mean height (CWH) and the relative abundance of microbiota among sampling sites. Two-way ANOVA was performed to evaluate the effects of altitude-associated gradients, soil depth, and their interaction on soil physicochemical properties and microbial abundance. Mantel tests assessed statistical associations between individual microbial α-diversity indices and individual vegetation characteristics. For each altitude, the analysis included the nine soil sampling quadrats and their nine spatially matched vegetation observations (n = 9). Euclidean distance matrices were calculated separately for Chao1 richness, Shannon diversity, Simpson diversity, and each vegetation variable. All statistical analyses were performed using SPSS Statistics (Version 26.0; IBM Corp., Armonk, NY, USA), with statistical significance defined as *p* < 0.05.

## 3. Results

### 3.1. Vegetation, Soil Characteristics Along Altitudinal Gradients and Soil Depths

The species richness and density were not different (*p* > 0.05) among the three altitudes, whereas greater (*p* < 0.05) AGB and CWH were observed at low-altitude than at mid- and high-altitude alpine ecosystems (Table 1). The contents of dry matter, crude fat, neutral detergent fiber and crude ash of the forage samples did not differ along the altitudes; however, the crude protein at high altitude was significantly lower (*p* < 0.05) than that at low altitude. Meanwhile, the acid detergent fiber at mid-altitude was higher (*p* < 0.05) than that at low and high altitudes; it did not differ between low and high altitudes. Regression analysis showed that the AGB (R^2^ = 0.21303, *p* = 0.0027) and CWH (R^2^ = 0.31685, *p* < 0.001) were negatively correlated with altitude (Figure S1).

Effects of altitude, soil depth and their interaction on soil physicochemical properties (including pH, SOC, NH_4_^+^-N, NO_3_^−^-N and EC) were analyzed, and there was an altitude × depth interaction (*p* < 0.05) for all of these indices (Table 2 and Table S1). The pH of the soil decreased (*p* < 0.05) as either the altitude or depth increased, with significantly lower pH at the 20–30 cm layer than at the 0–10 cm layer at the same altitude and significantly lower pH at higher altitude at each soil layer. The SOC at middle and high altitudes was greater (*p* < 0.05) than that at low altitude, while at each altitude, the SOC was almost not affected by soil depth at middle or high altitude. Significantly greater (*p* < 0.05) NH_4_^+^-N contents were observed at high altitude than at low and mid-altitude. NH_4_^+^-N contents in surface soil (0–10 cm) were higher (*p* < 0.05) than in deeper layers (20–30 cm) at low and mid-altitude, whereas the NH_4_^+^-N contents significantly increased (*p* < 0.05) with increasing soil layer at high altitude. Greater (*p* < 0.05) NO_3_^−^-N contents were detected at surface-layer soil of middle and high altitudes and in medium-layer soil at high altitude, but there was no difference (*p* > 0.05) among the deeper-layer samples (20–30 cm). At low altitude, the soil EC was significantly higher in the surface layer than in deeper layers; meanwhile, the EC in the surface soil at low altitude was significantly higher than that in mid- and high-altitude soil.

### 3.2. Soil Microbial Communities Along Altitudinal Gradients

#### 3.2.1. Summary of Collective Sequencing Data of Soil Microbiota

A total number of 8,740,234 raw reads were generated from 81 soil samples. After data processing, including filtering, quality control, assembling of paired-end reads, and removal of primers, chimeras, and low-confidence singletons, a total of 7,957,816 high-quality reads, with a high-quality sequence ratio of 91.1%, were used for data analysis. A total of 67,705 ASVs were identified by the analysis based on 97% nucleotide sequence identity among reads. There were 778 shared bacterial ASVs among different soil depths and altitudes; however, a great number of individual ASVs were observed in each soil layer among different altitudes (Figure S2). Further, principal component analysis (PCA) based on 16S rRNA amplicon sequence variants revealed variation in microbial community structure among different altitude groups. In several comparisons, samples showed partial separation along PC1 and PC2, indicating altitude-associated shifts in community composition, although some overlap among groups was observed (Figure S3). Effects of altitude, soil depth and their interaction on the α-diversity indices (including Chao 1, Shannon, and Simpson) of soil microbiota were analyzed, and no altitude × depth interactions were observed (Table S2). At low and high altitudes, different patterns for the Chao 1 index were observed. It increased (*p* < 0.05) with soil depth at low altitude, but it decreased with soil depth at high altitude; meanwhile, it significantly increased (*p* < 0.05) with altitude at the surface soil. The Shannon and Simpson indexes remained stable at different soil depths and altitudes.

Taxonomic composition of the soil bacteria by altitude and depth was compared on the basis of percentage mean relative abundance. Except for some unclassified phyla, Proteobacteria (average 21.5%) and Acidobacteria (average 20.8%) were the dominant phyla, followed by Actinobacteriota, Methylomirabilota and Chloroflexota. Similarly, UBA12499 and VBCG01 were the dominant genera after the identification of the top 10 species at the genus level.

#### 3.2.2. Differential Microbes

**Figure 1:**
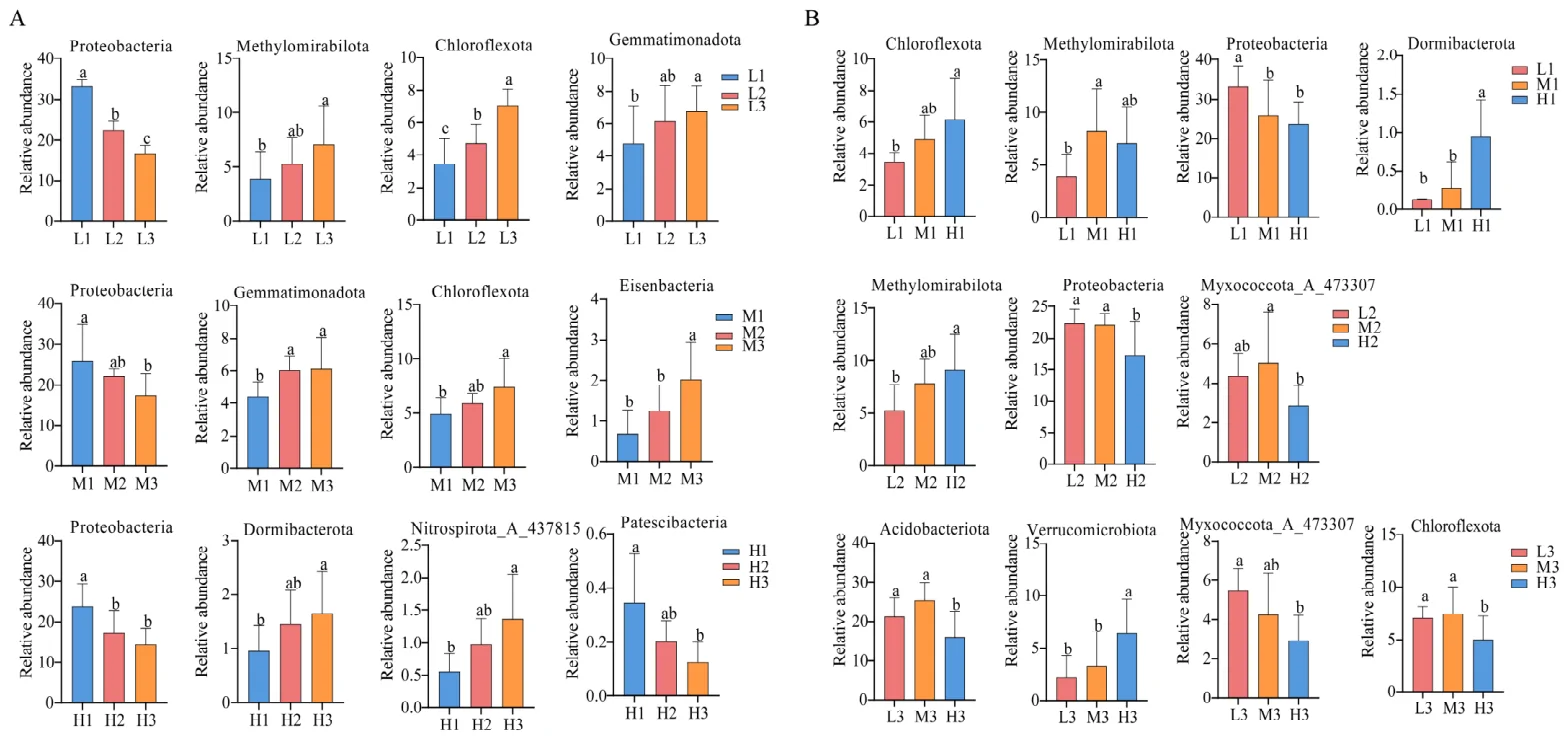
Differential soil microbes at the phylum level along soil depths (**A**) and altitudinal gradients (**B**) in alpine grasslands of Gannan prefecture. L1, L2, and L3 represent soil depths of 0–10 cm, 10–20 cm, and 20–30 cm in low altitudes; M1, M2, and M3 represent soil depths of 0–10 cm, 10–20 cm, and 20–30 cm in middle altitudes; and H1, H2, and H3 represent soil depths of 0–10 cm, 10–20 cm, and 20–30 cm in high altitudes. The same applies below. Different lowercase letters represent significant differences with *p* < 0.05.

The relative abundances of microbes at different soil depth of each altitude were compared, and differential microbes identified are shown in Figure 1A. Proteobacteria was the most dominant phylum at low-, mid- and high-altitudes, with its relative abundance significantly decreased (*p* < 0.05) as the soil depths increased at all the three altitudes. At low altitude, the relative abundance of Methylomirabilota was higher (*p* < 0.05) in the deeper layer (L3) than the surface layer (L1), and Desulfobacterota_B was higher (*p* < 0.05) in L3 and L2 than L1. In addition, the relative abundance of Nitrospirota_A_437815 was highest (*p* < 0.05) in L3, followed by L2 and L1 (*p* < 0.05). At middle altitude, the relative abundances of Chloroflexota, Gemmatimonadota and Eisenbacteria increased as the soil depth increased, with significantly greater (*p* < 0.05) abundance in M3 than in M1. At high altitude, the relative abundances of Nitrospirota_A_437815 and Dormibacterota were higher in the deeper layer (H3) than in the surface layer (H1); in contrast, Patescibacteria was higher (*p* < 0.05) in the surface layer (H1) than in the deeper layer (H3). At the genus level, the relative abundance of *VBCG01* was higher (*p* < 0.05) in the surface layer (L1, M1) than in the deeper layer (L3, M3), and *Usitatibacter* was higher (*p* < 0.05) in L1 and L2 than L3, and higher (*p* < 0.05) in H1 than H3 (Figure S4A).

#### 3.2.3. Metabolic Pathways

**Figure 2:**
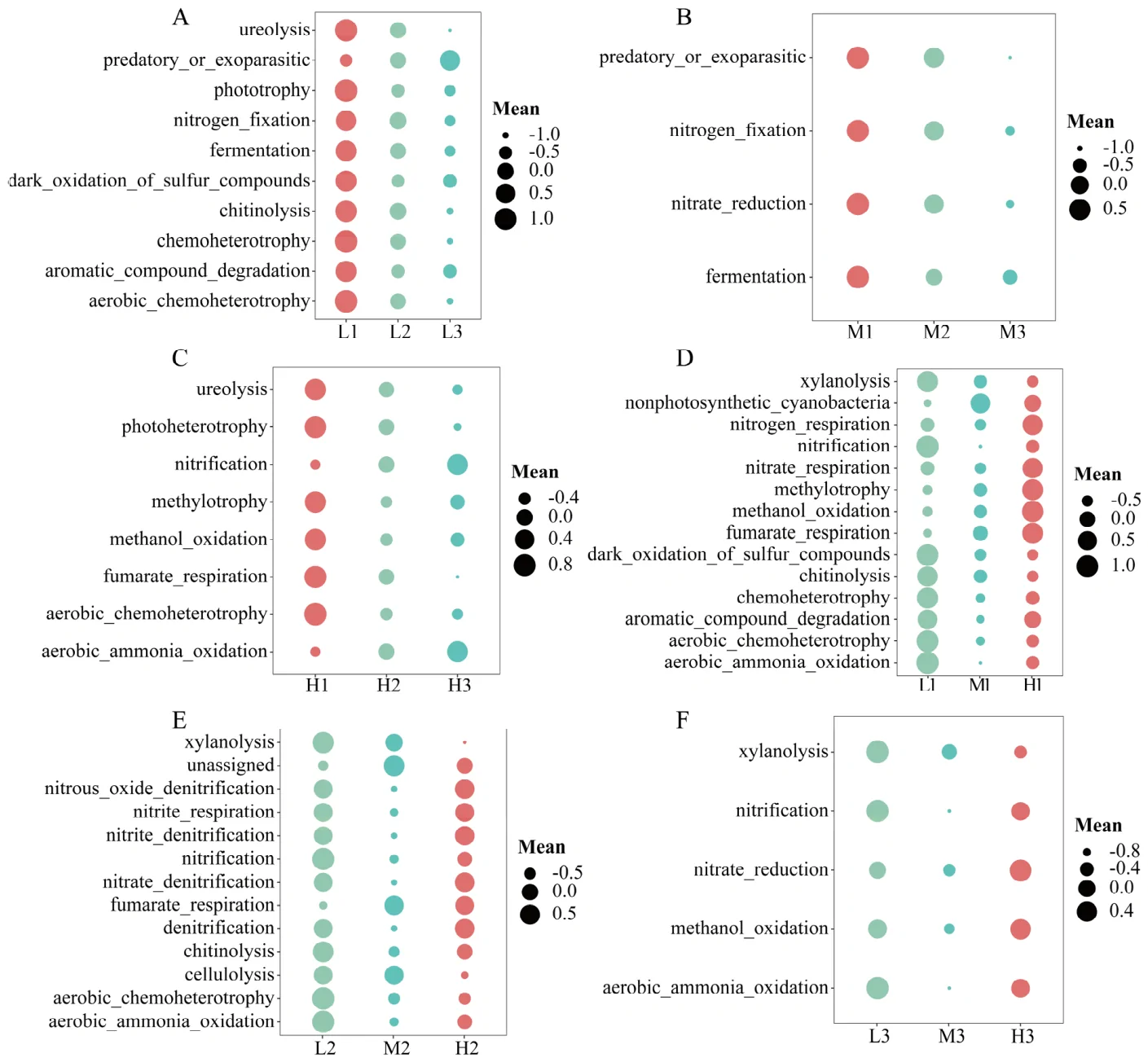
Differences in soil microbial function prediction at metabolic pathways. (**A**–**C**) represent the comparison of microbial metabolic pathways among different soil depths of the same altitude (low, middle and high altitude, respectively); (**D**–**F**) represent the comparison of microbial metabolic pathways at the same soil depth but different altitudes.

Based on FAPROTAX-predicted functional potentials, most soil microbial functions (including ureolysis, phototrophy, nitrogen fixation, fermentation, dark oxidation of sulfur compounds, chitinolysis, chemoheterotrophy, aromatic compound degradation and aerobic chemoheterotrophy) were higher (*p* < 0.05) in L1 than L2 or L3, but predatory or exoparasitic activity was lower (*p* < 0.05) in L1 than L3 (Figure 2). The enrichment function at mid-altitude was similar, with the four metabolic pathways in M1 higher (*p* < 0.05) than that in M3. At high altitude, nitrification and aerobic ammonia oxidation in H3 were higher (*p* < 0.05) than those in H1 (*p* < 0.05).

### 3.3. Soil Microbial Communities in Different Soil Depths

#### 3.3.1. Differential Microbes

The relative abundances of microbes at the same soil depth (0–10 cm, 10–20 cm, and 20–30 cm, respectively) but different altitudes were compared, with different microbes identified (Figure 1B). In surface soil (0–10 cm), the relative abundances of Chloroflexota and Dormibacterota were significantly higher at high altitude than at low or mid-altitude; whereas the Methylomirabilota and Nitrospirota-A-437815 had the highest abundance at mid-altitude. In the medium soil (10–20 cm), the relative abundances of Methylomirabilota and Dormibacterota were higher (*p* < 0.05) at high versus low altitude; while the relative abundances of Myxococota_A_473307 and Nitrospirota_A_437815 were greater at mid-altitude. In the deeper layer (20–30 cm), the relative abundance of Acidobacteriota was higher (*p* < 0.05) at low or mid-altitude than at high altitude; Eisenbacteria had the highest abundance at low altitude, and Verrucomicrobiota had the highest abundance at high altitude. At the genus level, the relative abundance of TA_21 was higher (*p* < 0.05) at low altitude than middle and high altitude in both the surface and the medium soil depths. In the medium soil depth, the relative abundances of UBA12499 and GWA2_73_35 increased with increasing altitude; VBCG01 was higher (*p* < 0.05) in M2 than L2. In deeper soil, the relative abundances of VBCG01, PSRF01, GWC2_73_18 and UBA11740 were higher at mid-altitude (Figure S4B).

#### 3.3.2. Metabolic Pathways

At 0~10 cm, soil microbial function, including xylanolysis, nitrification, dark oxidation of sulfur compounds, chitinolysis, chemoheterotrophy, aromatic compound degradation, aerobic chemoheterotrophy, and aerobic ammonia oxidation, was higher (*p* < 0.05) in L1 than M1 or H1, whereas nitrogen respiration, nitrate respiration, methylothrophy, methanol oxidation, and fumarate respiration were higher in H1 (*p* < 0.05) than L1. At 10–20 cm, nitrification, aerobic chemoheterotrophy and aerobic ammonium oxidation were higher (*p* < 0.05) in L2 than in M2 or H2. At 20–30 cm, the predominant metabolic pathways were enriched in xylanosis, nitrification, nitrate reduction, methanol oxidation and aerobic ammonia oxidation, with comparable abundances between L3 and H3 (Figure 2).

### 3.4. Associations Between Soil Properties, Vegetation Characteristics, and Microbial Communities

#### 3.4.1. Relationship Between the Soil Microbial Community, Soil Physicochemical Properties and Vegetation Characteristics

**Figure 3:**
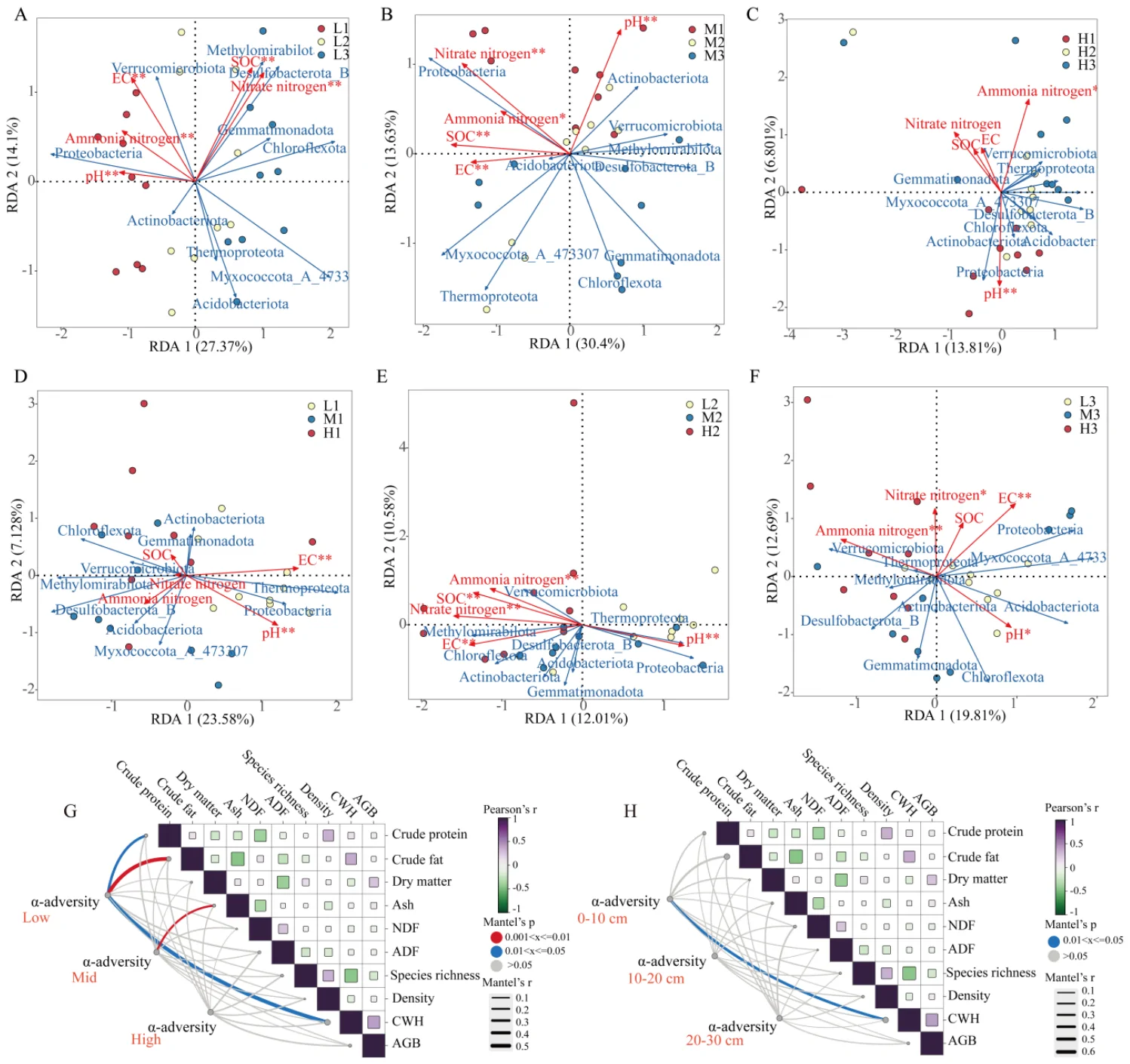
Correlation between soil microbes, soil physicochemical properties and vegetation characteristics analyzed using redundancy and Mantel analysis. Redundancy analysis (RDA) between soil microbiota and soil physicochemical properties in different soil depths at low (**A**), middle (**B**), and high altitude (**C**) and at the 0–10 cm (**D**), 10–20 cm (**E**), and 20–30 cm (**F**) soil depths of these three altitudes. *, 0.01 < *p* < 0.05; **, *p* ≤ 0.01. Correlation between α-diversity of soil microbiota and vegetation characteristics at different altitudes (**G**) and soil depths (**H**) based on Mantel test. SOC, soil organic carbon; EC, electrical conductivity; NDF, neutral detergent fiber; ADF, acid detergent fiber; CP, crude protein; AGB, aboveground biomass; CWH, community-weighted height. Percentages shown on the RDA axes indicate the proportion of total variance explained by each axis. Overall model statistics and permutation test results are provided in Table S3.

RDA analysis indicated that the associations between soil microbial community composition and soil physicochemical properties varied across altitudes (Figure 3A–C; Table S3). At low altitude, RDA1 and RDA2 explained 27.4% and 14.1% of the variation, respectively, yielding a cumulative explained proportion of 41.5% (Figure 3A). The overall model was significant (F = 3.961, *p* = 0.001), explaining 48.5% of the total variance (adjusted R^2^ = 0.363). At mid-altitude, the corresponding percentages were 30.4% and 13.6%, with a cumulative explained proportion of 44.0% (Figure 3B). The overall model explained 47.7% of the total variance (F = 3.826, *p* = 0.001; adjusted R^2^ = 0.352). At both altitudes, soil pH, SOC, EC, NH_4_^+^-N, and NO_3_^−^-N (*p* < 0.05) indicated significant associations with microbial community. In contrast, at high altitude, the first two axes jointly explained 20.6% of the total variance, whereas the overall model explained 26.2% and was not significant (F = 1.493, *p* = 0.104; adjusted R^2^ = 0.087). At low altitude, Mantel analysis indicated significant associations between the microbial community dissimilarity matrix and crude protein (Mantel *r* = 0.312, *p* = 0.014), crude fat (Mantel *r* = 0.493, *p* = 0.007), and community-weighted height (CWH; Mantel *r* = 0.505, *p* = 0.017) (Figure 3D). Among these variables, CWH showed the strongest association.

Along the soil depth gradient, the associations between soil physicochemical properties and microbial communities varied among soil layers (Figure 3E–G). The first two RDA axes jointly explained 30.7% of the total variation, and the overall model explained 39.1% (F = 2.696, *p* = 0.001; adjusted R^2^ = 0.246) on the surface layer (0–10 cm), with pH and EC showing significant associations. In the 10–20 cm layer, the first two axes jointly explained 22.6% of the variance, and the overall model explained 32.9% (F = 2.058, *p* = 0.003; adjusted R^2^ = 0.169). In the 20–30 cm layer, these proportions were 32.5% and 41.3%, respectively (F = 2.951, *p* = 0.001; adjusted R^2^ = 0.273). Significant associations with pH and NH_4_^+^-N were observed in the 10–20 cm layer and 20–30 cm layer (*p* < 0.05). pH was positively correlated with the relative abundance of Proteobacteria but negatively correlated with that of Verrucomicrobiota across all the three soil layers. At the 0–10 cm soil layer, Mantel analysis indicated a significant association between the microbial community dissimilarity matrix and community-weighted height (CWH; Mantel *r* = 0.426, *p* = 0.036) (Figure 3H).

#### 3.4.2. Soil Microbiota Co-Occurrence Patterns Along Altitudinal Gradients and Depths

**Figure 4:**
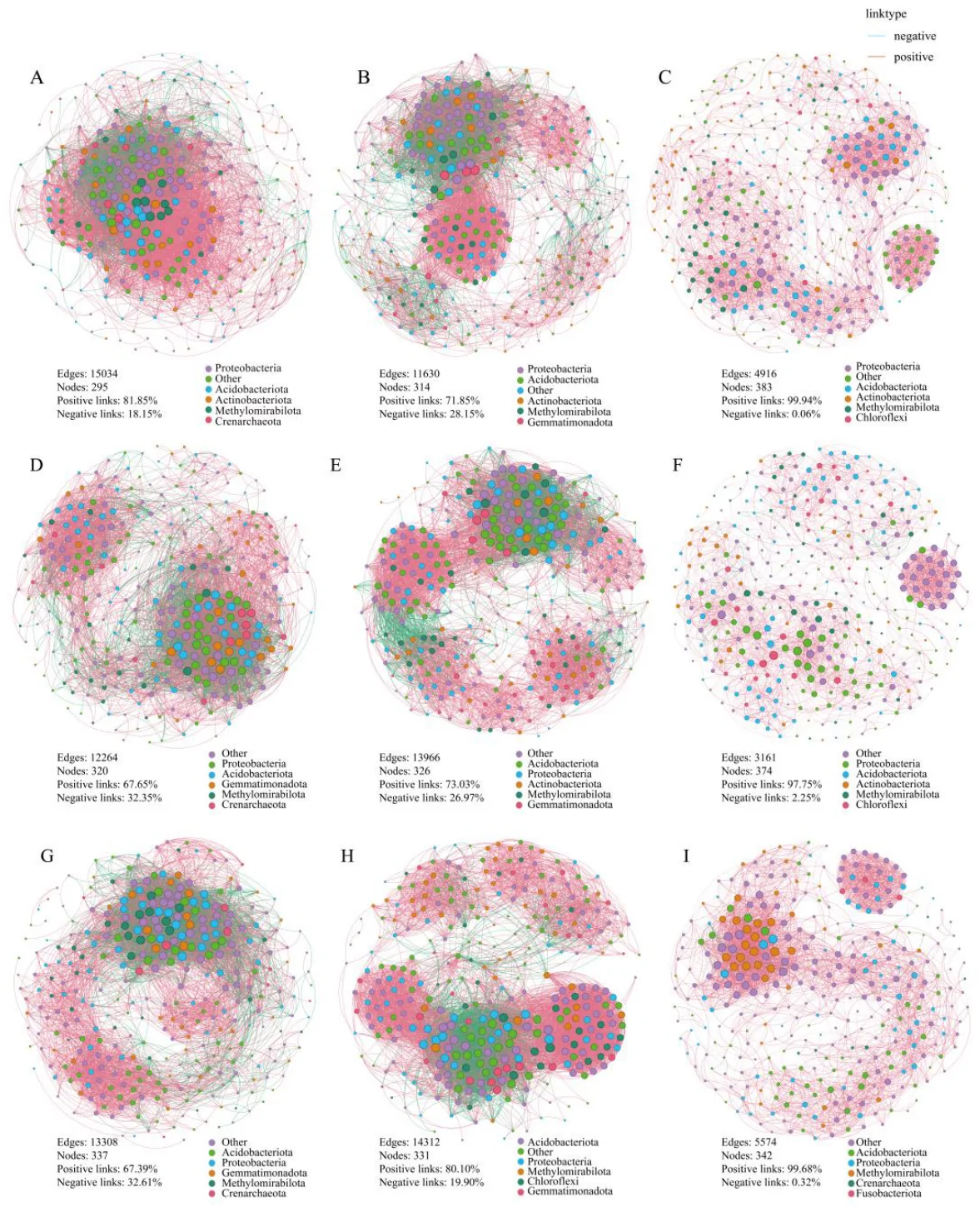
ASV-level soil microbial co-occurrence networks across altitude and soil-depth gradients. (**A**–**C**) represent the co-occurrence networks at 0–10, 10–20, and 20–30 cm layers of low altitude; (**D**–**F**) represent those at mid-altitude, while (**G**–**I**) represent those at high altitude. The nodes are colored by phylum level, and the size of each node is proportional to its degree. The line between each pair of nodes represents either positive (red) or negative (green) correlation with *p* < 0.05 and |r| > 0.85.

ASV-level co-occurrence networks, with nodes colored according to their phylum-level taxonomic affiliations, showed different topological properties among altitude × soil-depth groups (Figure 4; Table S4). At the phylum level, the soil microbial coexistence networks across all the altitude gradients exhibited significant depth-dependent changes (Figure 4). At low altitude (Figure 4A–C), despite an increase in the number of nodes, network connectivity decreased sharply with an increasing soil depth, as evidenced by a significant reduction in the number of edges, from 15,034 in the 0–10 cm layer to 11,630 in the 10–20 cm layer, and then to 4916 in the 20–30 cm layer. Similar depth-dependent simplification phenomena were also observed at mid-altitude (Figure 4D–F) and high-altitude (Figure 4G–I) regions, where the number of edges decreased from 12,264 and 13,308 in the surface and subsurface layers to 3161 and 5574 in the deeper layer. The proportion of positive connections increased with depth at low-altitude areas (from 81.9% to 99.9%), mid-altitude areas (from 67.7% to 97.8%) and high-altitude areas (from 67.4% to 99.7%).

Along the altitude gradient, the coexistence network structure within the same soil layer exhibited significant changes in interaction patterns. In the surface layer (0–10 cm), the proportion of positive correlations continued to decrease with increasing altitude, from 81.85% at low altitude to 67.7% at mid-altitude and 67.3% at high altitude; whereas the proportion of negative correlations increased accordingly. In the 10–20 cm soil layer, the proportion of positive and negative correlations was reversed. The proportion of negative correlations was lowest in the 20–30 cm soil layer (Figure 4; Table S4). High-altitude networks also exhibited lower average clustering coefficients and higher modularity than the corresponding low- and mid-altitude networks at each soil depth (Table S4).

## 4. Discussion

### 4.1. Altitude-Associated Environmental Gradients Shape Vegetation and Soil Properties

**Figure 5:**
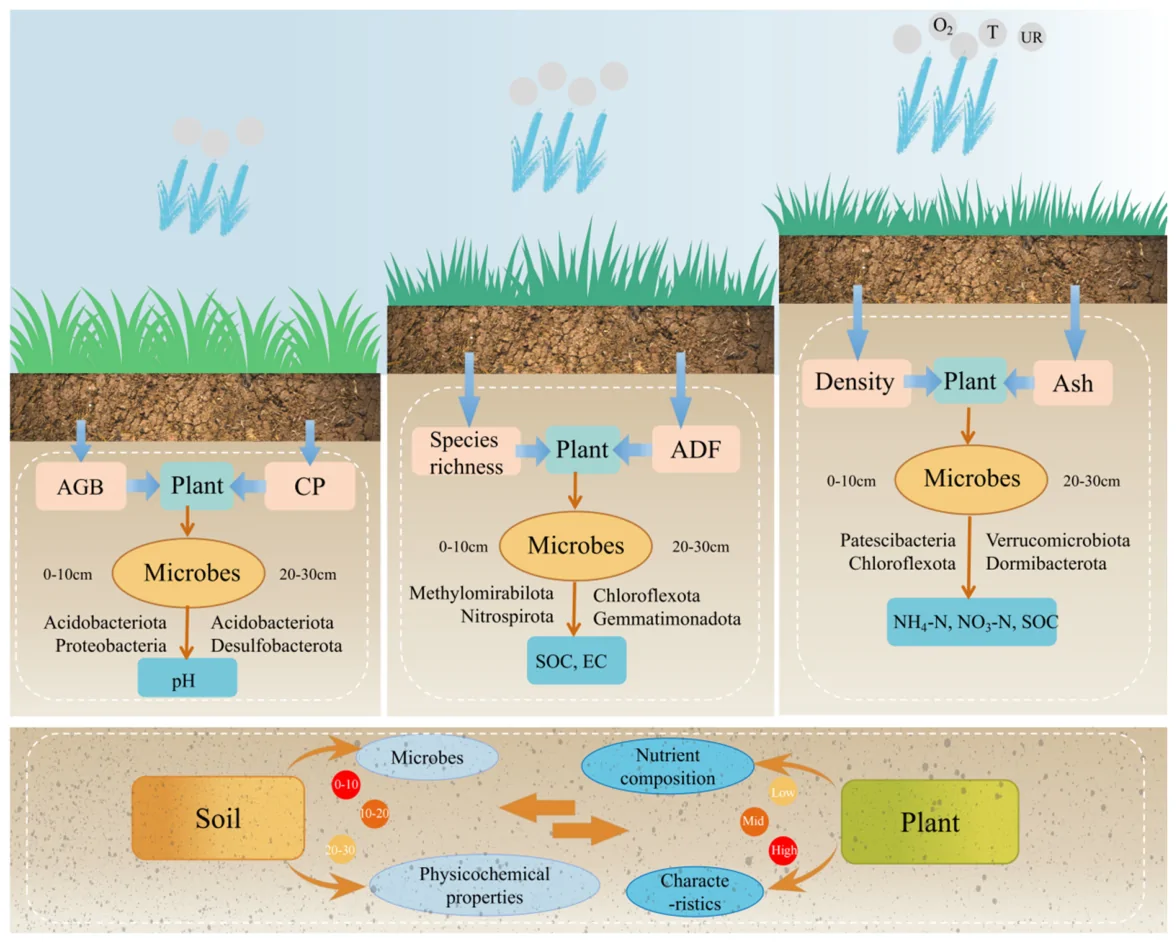
The mutual influence between soil and vegetation at different altitudes of alpine ecosystems on the Qinghai–Tibet Plateau, Gannan Tibetan Autonomous Prefecture, Gansu province, China. The length of the arrow represents the degree of influence of climate factors at different altitudes, while the circle represents climate factors. Only some climate factors are labeled. O_2_, oxygen; T, temperature; UR, ultraviolet radiation; AGB, aboveground biomass; CP, crude protein; ADF, acid detergent fiber; SOC, soil organic carbon; EC, electrical conductivity.

Altitude and soil depth are interdependent, and they jointly shape the characteristics of plateau ecosystems. As shown in Figure 5, the difference in vegetation characteristics influences the difference in soil physicochemical properties, which further reacts to the microbial community structure, and then forms the feedback mechanism of plant–soil interaction. The yield and quality of the pastures are constrained by vegetation types and environmental conditions, affecting their production and utilization efficiency [29]. This study found that the total AGB significantly decreased with increasing altitude, consistent with observations in the Xizang grassland [30] and the China Sanjiangyuan alpine meadow [10]. The meadow, adapted to high-altitude climates, is covered by low-growing herbaceous and cushion vegetation, leading to reduced biomass [31]. Additionally, linear fitting analysis also indicates that AGB and CWH significantly decrease with increasing altitude. This is because temperature decreases with increasing altitude, which strongly restricts plant growth and nitrogen uptake, thus resulting in higher AGB and CWH at low-altitude than at mid- to high-altitudes areas [24]. The significant decrease in crude protein content at high altitude suggests that the quality of pasture decreased with increasing altitude. Plant nutrient accumulation is associated with plant traits and soil physicochemical environment, and adequate soil nutrients are critical for enhancing forage productivity and quality [1,10].

Soil pH serves as a key link between plant growth, microbial activity, and nutrient availability. It not only modulates the morphology of chemical species and nutrient availability but also influences microbial growth and function [4]. In the current study, we confirmed that the soil pH continuously decreases with increasing altitude, which is consistent with the previous studies in mountain ecosystems [32]. This may be due to the fact that at higher altitudes, the environmental condition is relatively colder and wetter, alkaline cations in the soil are more easily leached out, and organic acids accumulate, which leads to soil acidification [33]. Meanwhile, the higher SOC content in mid -to high-altitude regions reflects a significant decrease in microbial decomposition rates under low temperature conditions, allowing SOC to gradually accumulate [34]. Furthermore, the depth-dependent patterns exhibited by NH_4_^+^-N and NO_3_^−^-N indicate that microbial-mediated nitrogen mineralization and nitrification processes undergo significant adjustments based on changes in environmental conditions and soil depth. This response is likely driven by soil pH and SOC content, which influence microbial activity and thus regulate nitrogen transformation rates [35]. From the perspective of soil layer influence, there are significant differences in physicochemical properties among soils of varying depths. Shallow soils typically exhibit higher organic matter input and more active microbial activity [30]. The forms of inorganic nitrogen present in the soil and its EC can reflect depth-specific microbial and geochemical processes, which are regulated by environmental gradients related to altitude [36].

### 4.2. Vertical Zonal Response of Soil Bacterial Diversity and Functional Communities to Altitude Gradients on the QTP

The microbial community structure differed markedly between the surface and subsurface soil layers, which is consistent with the previous findings that soil depth strongly influences microbial communities [37]. Similar to observations in the southern Loess Plateau, where abundant taxa show pronounced depth-dependent shifts in diversity and composition [38], we observed partial separation of microbial communities between surface and subsurface soil layers. These patterns indicate vertical differentiation in community composition across soil layers [6]. Across all the altitudes, Proteobacteria were dominant phyla but declined with increasing depth. Their ability to utilize readily decomposable substrates likely explains their surface enrichment [39]. In contrast, oligotrophic microorganisms like Chloroflexota and Dormibacterota adapted to resource-poor environments that increased with soil depth [40]. When comparing microbial patterns at the same soil depth across altitudinal gradients, high-altitude surface soil (0–10 cm) exhibited enrichment of Chloroflexota and Dormibacterota, which were relatively more abundant at high altitude. In the 10–20 cm layer, Methylomirabilota and Dormibacterota were more abundant at mid- and high altitudes, while Verrucomicrobiota dominated in the deeper soils (20–30 cm) at high altitude. These patterns are consistent with the reports that rising temperature reduces the abundance of several cold-adapted bacterial groups, particularly Chloroflexota, Dormibacterota, and Desulfobacterota [41,42]. Thus, the observed altitudinal patterns may be associated with climatic differences reported across altitudes.

Predicted functional potentials also varied across altitude and soil depth. In the low-altitude surface soils (L1), a suite of carbon- and nitrogen-related pathways, including ureolysis, nitrogen fixation, fermentation, chitin degradation, aromatic compound degradation, and aerobic heterotrophy, were significantly enriched, reflecting high organic matter inputs and active microbial turnover [43,44]. Similar enrichment occurred in mid-altitude surface soils (M1). In contrast, the deepest high-altitude soil group (H3) was associated with higher predicted relative abundances of functional groups related to nitrification and aerobic ammonia oxidation, suggesting an increased contribution of taxa potentially associated with chemolithotrophic metabolism. This functional profile aligns with the increased relative abundance of Nitrospirota (e.g., Nitrospirota_A_437815), a lineage containing canonical and complete ammonia oxidizers [45,46,47]. Together, these results suggest that carbon-dominated microbial metabolisms prevail in nutrient-rich surface soils, whereas nitrogen transformation pathways become increasingly important in deeper and high-altitude soils.

### 4.3. Associations Among Soil Properties, Vegetation Characteristics, and Microbial Communities

Along the altitudinal gradient, soil pH and nitrogen variables showed significant associations with microbial community composition in the RDA analyses. These observations are consistent with previous studies reporting associations between soil pH and bacterial community patterns in alpine grasslands [5]. Increasing altitude is typically associated with reduced temperature and plant biomass, which likely constrain SOC inputs while modifying nitrogen cycling processes, thereby strengthening the associations between pH and NH_4_^+^-N on microbial assemblages [48,49]. Accordingly, pH and NH_4_^+^-N remained significantly associated with the dominant bacterial taxa, including Actinobacteriota, Verrucomicrobiota, and Proteobacteria, particularly at high altitude [50]. Vegetation effects on microbial α-diversity also varied with altitude, with low-altitude diversity closely linked to vegetation quality indicators, suggesting an indirect a potential indirect association through soil nutrient conditions [51].

Associations between microbial communities and vegetation characteristics also varied across soil layers. Surface soils may receive greater inputs of plant residues and root-derived materials than deeper layer. In the surface soil (0–10 cm), plentiful inputs of organic matter from plant litter and root exudates favor fast-growing, copiotrophic taxa, such as Proteobacteria and Actinobacteria [52]. With increasing depth, declining labile carbon and a greater reliance on inorganic nitrogen likely favor oligotrophic and chemolithotrophic taxa that are adapted to low-carbon conditions, including Verrucomicrobiota and ammonia-oxidizing archaea; this is consistent with the patterns reported in previous studies [6,53]. At the 0–10 cm layer, microbial α-diversity showed a significant Mantel association with community-weighted height (CWH). However, labile carbon availability, oxygen conditions, and microbial functional activities were not directly measured in this study.

ASV-level co-occurrence network properties varied across soil depths and altitude zones. Specifically, the number of network edges sharply decreased from the surface to deep soil across low-, mid-, and high-altitude sites, consistent with previous findings that soil depth affects microbial interactions by changing resource availability and environmental conditions [54]. Research on peatlands shows that as soil depth increases, the positive interaction ratio and clustering coefficient rise while negative interactions decline, indicating that surface and deeper soils have distinct topological structures, with generally fewer coexisting connections in deeper soils [55]. Network topology also varied across altitude zones. High-altitude networks showed lower connectivity and higher modularity than the corresponding low- and mid-altitude networks, suggesting a more compartmentalized co-occurrence structure [56]. The proportions of positive and negative co-occurrence associations also differed among altitude zones and soil layers. These observations are broadly consistent with previous studies reporting altitudinal variation in microbial network topology and complexity [57]. However, the present correlation-based networks describe statistical co-occurrence patterns and do not demonstrate direct ecological interactions, such as cooperation, competition, or symbiosis. In addition, temperature, humidity, and resource availability were not included as explanatory variables in the network analyses; therefore, their potential contributions to the observed network patterns cannot be directly evaluated. Therefore, these co-occurrence patterns should be interpreted as statistical correlations that provide clues for potential ecological mechanisms.

## 5. Conclusions

Vegetation characteristics, soil physicochemical properties, bacterial community composition, predicted functional potentials, and ASV-level co-occurrence network properties varied across altitude zones and soil depths in the sampled alpine grasslands. AGB and CWH were negatively associated with altitude. On the surface soil layer, carbon-driven communities, with complex coexistence networks, are dominant, whereas the deeper layers exhibit simplified networks that are dominated by nutrient-poor and nitrogen cycling taxa. High-altitude networks showed lower connectivity and higher modularity than low- and mid-altitude networks, whereas depth-related network variation differed among altitudes. Together, these observations suggest links among vegetation, soil conditions, and bacterial community patterns but do not establish causal pathways or direct microbial interactions. Seasonal monitoring, manipulative experiments, and direct functional measurements are needed to evaluate the proposed mechanisms.

## Figures and Tables

**Table 1:** Vegetation characteristics and nutrient contents of natural pastures along altitude of Gannan alpine ecosystems located at the northeastern edge of Gannan alpine ecosystems.

Items	Altitudes ^1^		
	Low	Mid	High
Vegetation characteristics			
Species richness, species/m^2^	12 ± 0.7	11 ± 1.6	14 ± 1.3
Density, plants/m^2^	246 ± 25.6	229 ± 20.0	198 ± 22.8
Aboveground biomass, g/m^2^	249.8 ± 18.27 ^a^	173.8 ± 11.49 ^b^	180.0 ± 17.55 ^b^
Community weighted height ^2^, cm	19.0 ± 2.33 ^a^	12.3 ± 1.25 ^b^	9.4 ± 1.23 ^b^
Nutrient contents ^3^			
Crude protein, %	12.8 ± 0.85 ^a^	11.3 ± 0.55 ^ab^	10.1 ± 0.19 ^b^
Crude fat, %	2.8 ± 0.34	3.0 ± 0.21	2.7 ± 0.16
Dry matter, %	94.2 ± 0.12	93.8 ± 0.14	93.9 ± 0.13
Crude ash, %	11.9 ± 0.80	9.5 ± 0.73	12.2 ± 1.14
Neutral detergent fiber, %	38.1 ± 1.11	43.8 ± 2.06	39.5 ± 2.31
Acid detergent fiber, %	25.0 ± 1.08 ^b^	31.1 ± 1.79 ^a^	25.4 ± 1.18 ^b^

^1^ Low, 3000–3100 m; mid, 3300–3400 m; high, 3500–3600 m above sea level (the same for the tables below). This study was carried out in Gannan Tibetan Autonomous Prefecture, Gansu province, China. ^2^ Community-weighted height was calculated as: CWH = iSPi × Hi, where Pi is the percentage of abundance of functional group i, Hi is the mean plant height of functional group i, and S is the number of functional groups in the community. ^3^ The nutrient contents were measured on a dry matter basis. ^a,b^ indicates the values within a row with different superscripts differ (*p* < 0.05). Values are presented as mean ± SEM.

**Table 2:** Effects of altitudes and soil depths on physicochemical properties of soil collected from Gannan alpine ecosystems.

Items	Soil Depths, cm	Altitudes		
		Low	Mid	High
pH	0–10	7.6 ± 0.14 ^Aa^	7.1 ± 0.05 ^Ba^	6.8 ± 0.16 ^Ca^
	10–20	7.6 ± 0.14 ^Aa^	6.9 ± 0.10 ^Bb^	6.5 ± 0.16 ^Cb^
	20–30	7.2 ± 0.07 ^Ab^	6.8 ± 0.11 ^Bb^	6.4 ± 0.16 ^Cc^
Soil organic carbon	0–10	2.8 ± 0.33 ^Bb^	4.6 ± 1.22 ^A^	4.6 ± 1.65 ^A^
	10–20	3.5 ± 0.59 ^Ba^	3.8 ± 0.64 ^B^	6.0 ± 1.89 ^A^
	20–30	3.2 ± 0.37 ^Bab^	4.5 ± 1.22 ^A^	4.8 ± 0.44 ^A^
Ammonia nitrogen	0–10	22.4 ± 7.22 ^Ba^	30.6 ± 7.26 ^Aa^	24.8 ± 8.21 ^ABc^
	10–20	25.1 ± 6.19 ^Ba^	27.9 ± 7.45 ^Ba^	48.4 ± 11.23 ^Ab^
	20–30	7.0 ± 3.90 ^Bb^	14.4 ± 5.10 ^Bb^	84.5 ± 12.28 ^Aa^
Nitrate nitrogen	0–10	4.2 ± 0.94 ^Bb^	17.1 ± 8.55 ^Aa^	12.9 ± 10.85 ^A^
	10–20	11.0 ± 4.25 ^Bab^	10.3 ± 1.02 ^Bab^	18.0 ± 9.79 ^A^
	20–30	15.1 ± 11.07 ^a^	9.4 ± 8.05 ^b^	18.4 ± 9.14
Electrical conductivity	0–10	1.1 ± 0.03 ^Aa^	0.60 ± 0.29 ^B^	0.53 ± 0.45 ^B^
	10–20	0.63 ± 0.16 ^b^	0.83 ± 0.08	1.0 ± 0.80
	20–30	0.68 ± 0.35 ^b^	0.93 ± 0.73	0.75 ± 0.32

Different uppercase letters represent the differences among the same soil depth but different altitudes; while different lowercase letters represent the differences among different soil depths at the same altitude.

## Data Availability

The data presented in this study are openly available in NCBI Sequence Read Archive (SRA) at https://www.ncbi.nlm.nih.gov/bioproject/PRJNA1208410 (accessed on 5 January 2025), reference number PRJNA1208410.

## Supplementary Materials

The following supporting information can be downloaded at https://www.mdpi.com/article/10.3390/biology15181663/s1, Table S1. The effects of altitude and soil depth on soil physicochemical properties; Table S2. Effects of altitudes and soil depths on α-diversity of microbial community of soil collected from Gannan Alpine Meadow; Table S3. Summary of redundancy analysis (RDA) model statistics for different altitudes and soil depths; Table S4. Topological properties of bacterial co-occurrence networks across three altitude zones and three soil depths; Figure S1. Linear regression analysis between vegetation characteristics and altitude gradient. (A) aboveground biomass; (B) species richness; (C) community weighted height; (D) density; Figure S2. Venn diagram displaying shared and individual bacterial ASVs in different soil depths at low, middle and high altitude. L1, L2, L3, M1, M2, M3, H1, H2, and H3 represent soil depths of 0–10, 10–20 and 20–30 cm at low, middle and high altitude, respectively; the same applies to the figures below; Figure S3. Principal component analysis (PCA) at different soil depths and altitudes. (A, B and C) represent PCA plots among different soil depths of low, middle and high altitude, respectively; (D, E and F) represent PCA plots at the same soil depth but different altitudes; Figure S4. Differential microbes at genus levels along soil depths (A) and altitudinal gradients (B). L1, L2, L3, M1, M2, M3, H1, H2, and H3 represent soil depths of 0–10, 10–20 and 20–30 cm at low, middle and high altitude, respectively. Different lowercase letters represent significant differences with *p* < 0.05.

## References

[1] Philippot L., Chenu C., Kappler A., Rillig M.C., Fierer N. (2023). The interplay between microbial communities and soil properties. Nat. Rev. Microbiol..

[2] Falkowski P.G., Fenchel T., Delong E.F. (2008). The microbial engines that drive earth’s biogeochemical cycles. Science.

[3] Körner C. (2007). The use of ‘altitude’ in ecological research. Trends Ecol. Evol..

[4] Rousk J., Bååth E., Brookes P.C., Lauber C.L., Lozupone C., Caporaso J.G., Knight R., Fierer N. (2010). Soil bacterial and fungal communities across a pH gradient in an arable soil. ISME J..

[5] Chen B., Jiao S., Luo S., Ma B., Qi W., Cao C., Zhao Z., Du G., Ma X. (2021). High soil pH enhances the network interactions among bacterial and archaeal microbiota in alpine grasslands of the Tibetan Plateau. Environ. Microbiol..

[6] Eilers K.G., Debenport S., Anderson S., Fierer N. (2012). Digging deeper to find unique microbial communities: The strong effect of depth on the structure of bacterial and archaeal communities in soil. Soil Biol. Biochem..

[7] Ji L., Shen F., Liu Y., Yang Y., Wang J., Purahong W., Yang L. (2022). Contrasting altitudinal patterns and co-occurrence networks of soil bacterial and fungal communities along soil depths in the cold-temperate montane forests of China. CATENA.

[8] Xu Z., Wang Y., Sun D., Li H., Dong Y., Wang Z., Wang S. (2022). Soil nutrients and nutrient ratios influence the ratios of soil microbial biomass and metabolic nutrient limitations in mountain peatlands. CATENA.

[9] Wang Z., Yang G., Yi S., Chen S., Wu Z., Guan J., Zhao C., Zhao Q., Ye B. (2012). Effects of environmental factors on the distribution of plant communities in a semi-arid region of the Qinghai-Tibet Plateau. Ecol. Res..

[10] Chen D., Li Q., Liu Z., He F.Q., Chen X., Xu S.X., Zhao X.Q., Zhao L. (2020). Variations of forage yield and nutrients with altitude gradients and their influencing factors in slpine meadow of Sanjiangyuan, China. J. Soil Sci. Plant Nutr..

[11] Dlamini P., Sekhohola Dlamini L.M., Cowan A.K. (2023). Soil-microbial interactions. Front. Microbiol..

[12] Zhang R., Tian X., Xiang Q., Penttinen P., Gu Y. (2022). Response of soil microbial community structure and function to different altitudes in arid valley in Panzhihua, China. BMC Microbiol..

[13] Yu J., Li S., Sun X., Zhou W., He L., Zhao G., Chen Z., Bai X., Zhang J. (2023). The impact and determinants of mountainous topographical factors on soil microbial community characteristics. Microorganisms.

[14] AOAC International (2005). Official Methods of Analysis.

[15] Li H., Li J., He Y.L., Li S.J., Liang Z.S., Peng C.H., Polle A., Luo Z.B. (2013). Changes in carbon, nutrients and stoichiometric relations under different soil depths, plant tissues and ages in black locust plantations. Acta Physiol. Plant..

[16] Ren C., Zhang W., Zhong Z., Han X., Yang G., Feng Y., Ren G. (2018). Differential responses of soil microbial biomass, diversity, and compositions to altitudinal gradients depend on plant and soil characteristics. Sci. Total Environ..

[17] Fatima F., Pathak N., Rastogi Verma S. (2014). An improved method for soil DNA extraction to study the microbial assortment within rhizospheric region. Mol. Biol. Int..

[18] Li Q., Wang P., Zou C., Ge F., Li F., Liu Y., Zhang D., Tian J. (2023). Dynamics of dominant rhizospheric microbial communities responsible for trichlorfon absorption and translocation in maize seedlings. J. Hazard. Mater..

[19] Bolyen E., Rideout J.R., Dillon M.R., Bokulich N.A., Abnet C.C., Al-Ghalith G.A., Alexander H., Alm E.J., Arumugam M., Asnicar F. (2019). Reproducible, interactive, scalable and extensible microbiome data science using QIIME 2. Nat. Biotechnol..

[20] Callahan B.J., McMurdie P.J., Rosen M.J., Han A.W., Johnson A.J.A., Holmes S.P. (2016). DADA2: High-resolution sample inference from Illumina amplicon data. Nat. Methods.

[21] Bokulich N.A., Kaehler B.D., Rideout J.R., Dillon M., Bolyen E., Knight R., Huttley G.A., Caporaso J.G. (2018). Optimizing taxonomic classification of marker-gene amplicon sequences with QIIME 2’s q2-feature-classifier plugin. Microbiome.

[22] Quast C., Pruesse E., Yilmaz P., Gerken J., Schweer T., Yarza P., Peplies J., Glöckner F.O. (2013). The SILVA ribosomal RNA gene database project: Improved data processing and web-based tools. Nucleic Acids Res..

[23] Dixon P. (2003). Vegan, a package of R functions for community ecology. J. Veg. Sci..

[24] Yoshiki V., Pirrung M., Gonzalez A., Knight R. (2013). Emperor: A tool for visualizing high-throughput microbial community data. GigaScience.

[25] Louca S., Parfrey L.W., Doebeli M. (2016). Decoupling function and taxonomy in the global ocean microbiome. Science.

[26] Barberán A., Bates S., Casamayor E., Fierer N. (2012). Using network analysis to explore co-occurrence patterns in soil microbial communities. ISME J..

[27] Quan Q., He N., Zhang R., Wang J., Luo Y., Ma F., Pan J., Wang R., Liu C., Zhang J. (2024). Plant height as an indicator for alpine carbon sequestration and ecosystem response to warming. Nat. Plants.

[28] Sun L., Sun J., Wu J., Du Z., Chen Y., Wang Y., Liu M., Chen W., Liang E. (2023). Plant community traits and functions mediate the biomass trade-off of alpine grasslands along precipitation gradients on the Tibetan Plateau. J. Plant Ecol..

[29] Shi Y., Ma Y., Ma W., Liang C., Zhao X., Fan J., He J. (2012). Large scale patterns of forage yield and quality across Chinese grasslands. Chin. Sci. Bull..

[30] Bhandari J., Zhang Y.J. (2019). Effect of altitude and soil properties on biomass and plant richness in the grasslands of Tibet, China, and Manang District, Nepal. Ecosphere.

[31] Pérez C.A., Frangi J.L. (2000). Grassland biomass dynamics along an altitudinal gradient in the Pampa. J. Range Manag..

[32] Shamsher A., Hussain I., Hussain S., Hussain A., Ali H., Ali M. (2019). Effect of Altitude on Forest Soil Properties at Northern Karakoram. Eurasian Soil Sci..

[33] Wang B., Li H., Li X., Liang X., Wang L., Zhan F., He Y., Si Z., He S. (2026). Response of Soil Organic Carbon Components in Pinus yunnanensis Stand to Altitude Variation. Agronomy.

[34] Liu Q., Zhang A., Li X., Yin J., Zhang Y., Sun O., Jiang Y. (2025). Changes in soil organic and inorganic carbon with elevation in a dry alpine rangeland of the northern Qinghai Tibet Plateau. Biogeosciences.

[35] Cheng Y., Wang J., Mary B., Zhang J., Cai Z., Chang S. (2013). Soil pH has contrasting effects on gross and net nitrogen mineralizations in adjacent forest and grassland soils in central Alberta, Canada. Soil Biol. Biochem..

[36] Husson O. (2012). Redox potential (Eh) and pH as drivers of soil/plant/microorganism systems: A transdisciplinary overview pointing to integrative opportunities for agronomy. Plant Soil.

[37] Chu H., Sun H., Tripathi B., Adams J., Huang R., Zhang Y., Shi Y. (2016). Bacterial community dissimilarity between the surface and subsurface soils equals horizontal differences over several kilometers in the western Tibetan Plateau. Environ. Microbiol..

[38] Peng Z., Wang Z., Liu Y., Yang T., Chen W., Wei G., Jiao S. (2021). Soil phosphorus determines the distinct assembly strategies for abundant and rare bacterial communities during successional reforestation. Soil Ecol. Lett..

[39] Jiao N., Luo T., Chen Q., Zhao Z., Xiao X., Liu J., Jian Z., Xie S., Thomas H., Herndl G.J. (2024). The microbial carbon pump and climate change. Nat. Rev. Microbiol..

[40] Liu J., Peng Z., Tu H., Qiu Y., Liu Y., Li X., Gao H., Pan H., Chen B., Liang C. (2024). Oligotrophic microbes are recruited to resist multiple global change factors in agricultural subsoils. Environ. Int..

[41] Lin D., Du S., Zhao Z., Zhang T., Wang L., Zhang Q., Zhou S.Y.D., Graham D.W., Tissue D.T., Zhu D. (2025). Climate warming fuels the global antibiotic resistome by altering soil bacterial traits. Nat. Ecol. Evol..

[42] Guo X., Yuan M., Lei J., Shi Z., Zhou X., Li J., Deng Y., Yang Y., Wu L., Luo Y. (2022). Climate warming restructures seasonal dynamics of grassland soil microbial communities. mLife.

[43] Fierer N., Bradford M.A., Jackson R.B. (2007). Toward an ecological classification of soil bacteria. Ecology.

[44] Han S., Luo X., Hao X., Ouyang Y., Zeng L., Wang L., Wen S., Wang B., Van Nostrand J.D., Chen W. (2021). Microscale heterogeneity of the soil nitrogen cycling microbial functional structure and potential metabolism. Environ. Microbiol..

[45] Bahram M., Espenberg M., Pärn J., Lehtovirta-Morley L., Anslan S., Kasak K., Kõljalg U., Liira J., Maddison M., Moora M. (2022). Structure and function of the soil microbiome underlying N2O emissions from global wetlands. Nat. Commun..

[46] Hamonts K., Clough T.J., Stewart A., Clinton P.W., Richardson A.E., Wakelin S.A., O’Callaghan M., Condron L.M. (2013). Effect of nitrogen and waterlogging on denitrifier gene abundance, community structure and activity in the rhizosphere of wheat. FEMS Microbiol. Ecol..

[47] Sun S., Lu C., Liu J., Williams M.A., Yang Z., Gao Y., Hu X. (2020). Antibiotic resistance gene abundance and bacterial community structure in soils altered by ammonium and nitrate concentrations. Soil Biol. Biochem..

[48] Wang J., Cao P., Hu H., Li L., Han L., Zhang L., Zheng Y., Hu J. (2015). Altitudinal distribution patterns of soil bacterial and archaeal communities along Mt. Shegyla on the Tibetan Plateau. Microbial. Ecol..

[49] Yang Y., Gao Y., Wang S., Xu D., Yu H., Wu L., Lin Q., Hu Y., Li X., He Z. (2014). The microbial gene diversity along an elevation gradient of the Tibetan grassland. ISME J..

[50] Xiang J., Gu J., Wang G., Bol R., Yao L., Fang Y., Zhang H. (2024). Soil pH controls the structure and diversity of bacterial communities along elevational gradients on Huangshan, China. Eur. J. Soil Biol..

[51] Liu L., Zhu K., Wurzburger N., Zhang J. (2020). Relationships between plant diversity and soil microbial diversity vary across taxonomic groups and spatial scales. Ecosphere.

[52] Liao J., Dou Y., Yang X., An S. (2023). Soil microbial community and their functional genes during grassland restoration. J. Environ. Manag..

[53] Lu X., Seuradge B.J., Neufeld J.D. (2017). Biogeography of soil Thaumarchaeota in relation to soil depth and land usage. FEMS Microbiol. Ecol..

[54] Mundra S., Kjønaas O.J., Morgado L.N., Krabberød A.K., Ransedokken Y., Kauserud H. (2021). Soil depth matters: Shift in composition and inter-kingdom co-occurrence patterns of microorganisms in forest soils. FEMS Microbiol. Ecol..

[55] Zhao M., Wang M., Zhao Y., Hu N., Qin L., Ren Z., Wang G., Jiang M. (2022). Soil microbial abundance was more affected by soil depth than the altitude in peatlands. Front. Microbiol..

[56] Chen Z., Xu Y., Wang X., Ma T., Liu Y., Qin X., Zhang W., Chen T., Liu G., Zhang B. (2025). Altitudinal patterns of bacterial communities across soil layers in the alpine meadows of the Qinghai-Tibet Plateau. Ecol. Indic..

[57] Chen W., Wang J., Chen X., Meng Z., Xu R., Duo D., Zhang J., He J., Wang Z., Chen J. (2022). Soil microbial network complexity predicts ecosystem function along elevation gradients on the Tibetan Plateau. Soil Biol. Biochem..

