# Supplementary material for: Contrasting Patterns of Soil Microbial Communities, Physicochemical Properties and Vegetation Characteristics Along Altitude and Soil Depths in an Alpine Ecosystem
Source: Biology (Basel). 2026 Sep 19;15(18):1663. doi: 10.3390/biology15181663 (PMC13604108; doi:10.3390/biology15181663)
Supplement: Supplementary file 1 [file biology-15-01663-s001.zip › biology-4539782-supplementary.pdf]

Table S1. The effects of altitude and soil depth on soil physicochemical properties

Soil physicochemical properties	Altitude[1]		Depth		Altitude × Depth	
	F-value[2]	*P*-value	F-value	*P*-value	F-value	*P*-value
pH	307.004	< 0.001	44.733	<0.001	3.653	0.009
SOC	19.494	<0.001	0.902	0.410	2.647	0.040
Ammonia nitrogen	124.162	<0.001	9.369	0.002	61.567	<0.001
Electrical conductivity	0.035	0.965	0.311	0.733	3.103	0.021
Nitrate nitrogen	3.892	0.025	0.808	0.450	3.182	0.018

[1]Altitude, effects of altitude on soil physicochemical properties and microbiota; Depth, effects of depth on soil physicochemical properties and microbiota; Altitude × Depth, interaction of altitude and soil depth on soil physicochemical properties and microbiota.

[2]F-value is the value of F-test;

Table S2. Effects of altitudes and soil depths on α-diversity of microbial community of soil collected from Gannan Alpine Meadow

Items	Soil depths, cm	Altitudes		
		Low	Mid	High
Chao 1	0-10	2471 ± 149^{Bb}	2832 ± 276^{A}	2906 ± 226^{Aa}
	10-20	2684 ± 247^{a}	2712 ± 204	2734 ± 194^{ab}
	20-30	2827 ± 108^{Aa}	2659 ± 331^{AB}	2576 ± 184^{Bb}
Shannon	0-10	10.29 ± 0.427	10.25 ± 0.278	10.04 ± 1.109
	10-20	9.98 ± 0.525	10.18 ± 0.162	9.80 ± 0.947
	20-30	10.24 ± 0.231^{A}	10.14 ± 0.324^{AB}	9.29 ± 1.479^{B}
Simpson	0-10	1.00 ± 0.001	1.00 ± 0.001	1.00 ± 0.008
	10-20	0.99 ± 0.01	1.00 ± 0.001	0.99 ± 0.009
	20-30	1.00 ± 0.002	1.00 ± 0.001	0.98 ± 0.029

[1] *P* value for soil depth and altitude interaction on α-diversity of microbial community.

Different uppercase letters represent the differences among the same soil depth but different altitudes; while different lowercase letters represent the differences among different soil depths at the same altitude.

Table S3 Summary of redundancy analysis (RDA) model statistics for different altitudes and soil depths.

Panel	Sample group	Model df	Residual df	F	P	R^2	Adjusted R^2
A	Low altitude	5	21	3.961	0.001	0.4853	0.3628
B	Mid-altitude	5	21	3.826	0.001	0.4767	0.3521
C	High altitude	5	21	1.493	0.104	0.2622	0.0866
E	0–10 cm	5	21	2.696	0.001	0.3910	0.2460
F	10–20 cm	5	21	2.058	0.003	0.3288	0.1690
G	20–30 cm	5	21	2.951	0.001	0.4127	0.2729

Notes: df, degrees of freedom. F and P were obtained from permutation tests of the overall RDA models. R^2 represents the proportion of total variance explained by all constrained axes. Adjusted R^2 was calculated for each RDA model.

Table S4 Topological properties of bacterial co-occurrence networks across three altitude zones and three soil depths.

Network metric	L1	L2	L3	M1	M2	M3	H1	H2	H3
Similarity threshold (St)	0.85	0.85	0.85	0.85	0.85	0.85	0.85	0.85	0.85
Number of edges	295	320	337	314	326	331	383	374	342
Number of links:	15034	12264	13308	11630	13966	14312	4916	3161	5574
Number of positive:	12306	8296	8968	8356	10200	11464	4913	3090	5556
Number of negative:	2728	3968	4340	3274	3766	2848	3	71	18
Positive edges (%):	81.85%	67.65%	67.39%	71.85%	73.03%	80.10%	99.94%	97.75%	99.68%
Negative edges (%):	18.15%	32.35%	32.61%	28.15%	26.97%	19.90%	0.06%	2.25%	0.32%
Average degree:	101.925	76.650	78.979	74.076	85.681	86.477	25.671	16.904	32.596
Average clustering coefficient:	0.636	0.644	0.636	0.663	0.673	0.677	0.557	0.522	0.588
Average path length:	2.760	2.979	2.950	2.902	2.854	2.852	4.789	4.532	3.864
Modularity:	0.244	0.452	0.414	0.504	0.569	0.574	0.717	0.740	0.644

Note: L, M, and H represent low-, mid-, and high-altitude sites, respectively. The numbers 1, 2, and 3 indicate soil depths of 0–10, 10–20, and 20–30 cm, respectively. Each network was constructed using nine composite soil samples from the corresponding elevation–depth combination. Nodes represent amplicon sequence variants (ASVs), and edges represent Spearman correlations meeting the thresholds of $|r| > 0.85$ and false discovery rate (FDR)-adjusted $P < 0.05$. Positive and negative edges indicate positive and negative correlations, respectively.

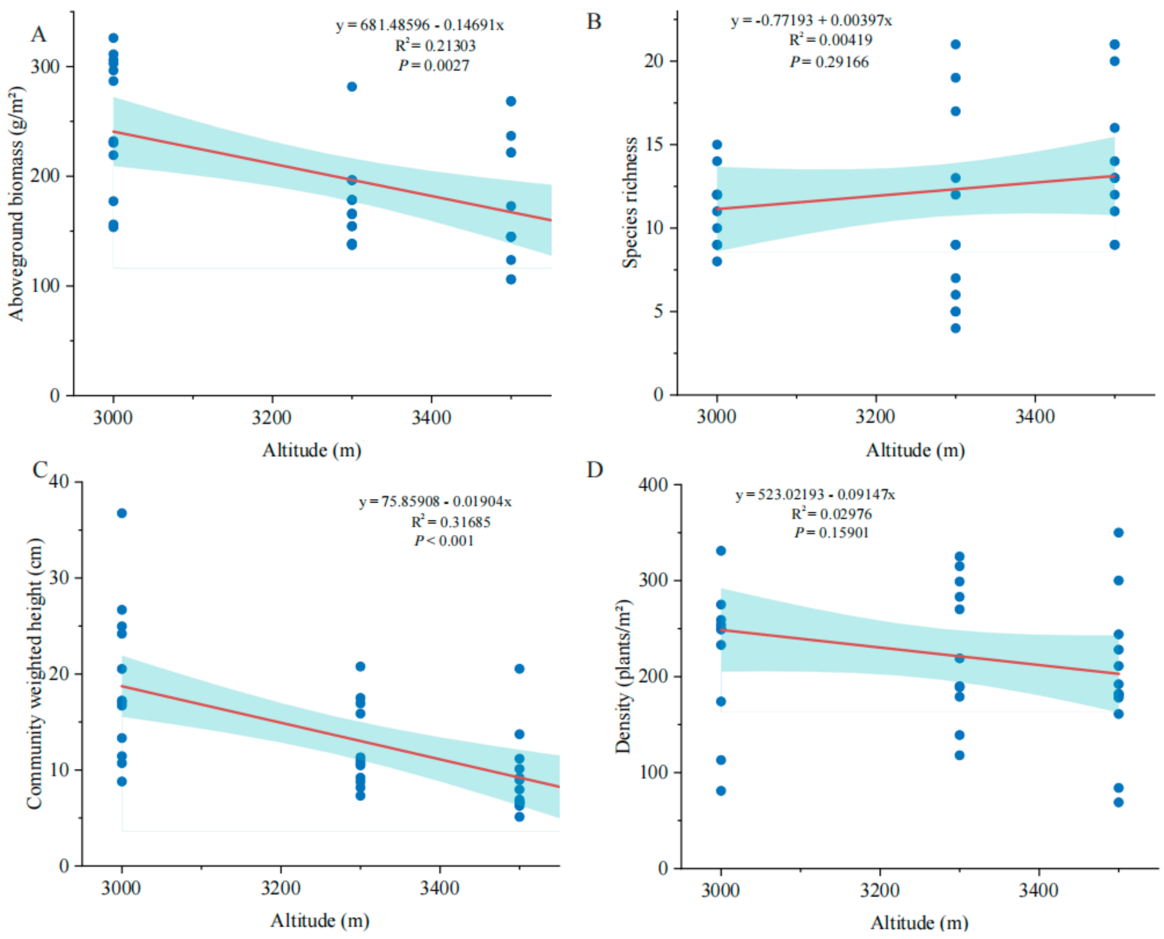

Figure S1 Linear regression analysis between vegetation characteristics and altitude gradient. A, aboveground biomass; B, species richness; C, community weighted height; D, density.

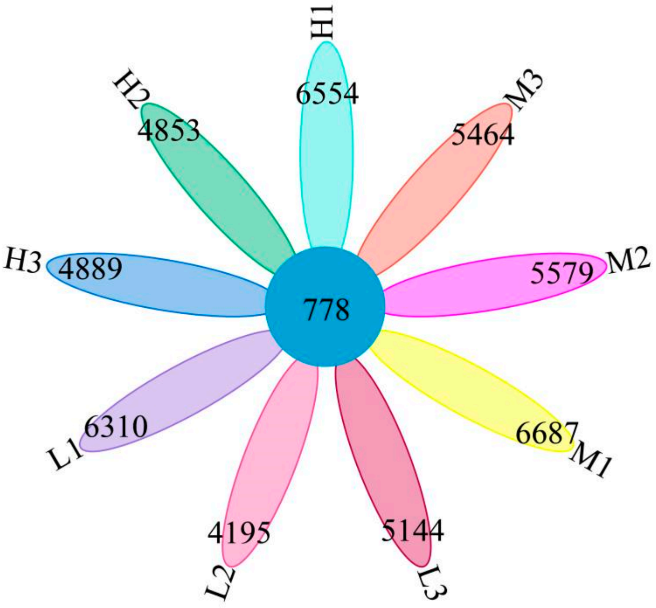

Figure S2 Venn diagram displaying shared and individual bacterial ASVs in different soil depths at low-, mid- and high-altitude. L1, L2, L3, M1, M2, M3, H1, H2, H3 represent soil depth of 0-10, 10-20 and 20-30 cm at low-, mid- and high-altitude, respectively; the same for the figures below.

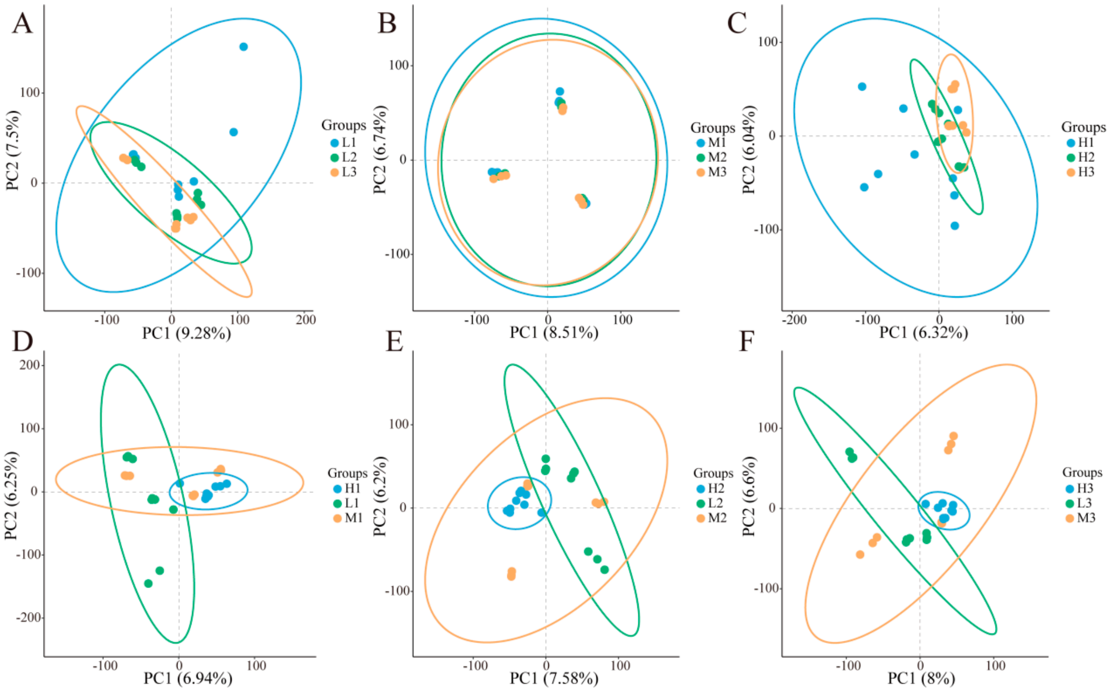

Figure S3 Principal component analysis (PCA) at different soil depths and altitudes. A, B and C represent PCA plot among different soil depths of low-, mid- and high-altitude, respectively; D, E and F represent PCA plot at the same soil depth but different altitude.

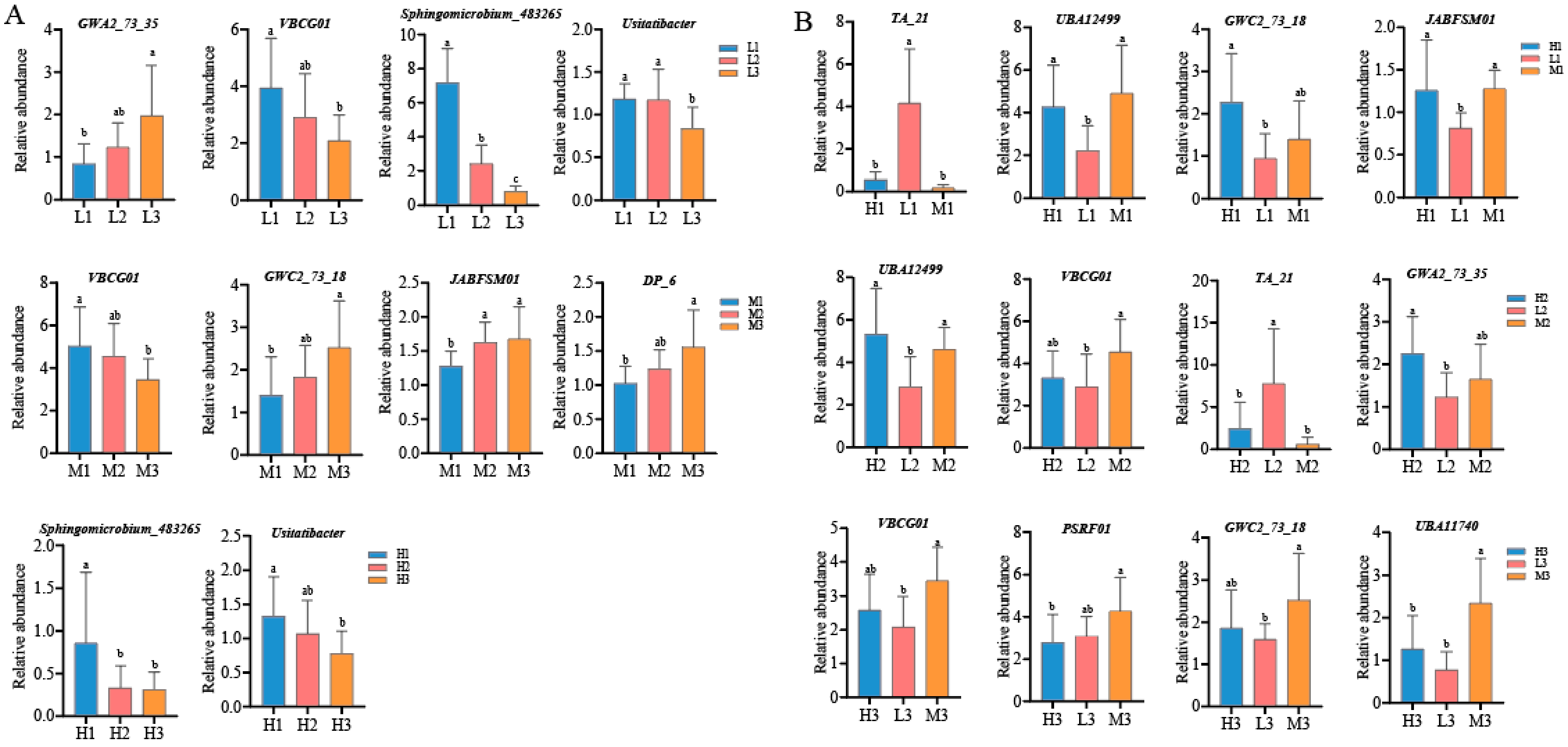

Figure S4 Differential microbes at genus levels along soil depths (A) and altitudinal gradients (B). L1, L2, L3, M1, M2, M3, H1, H2, H3 represent soil depth of 0-10, 10-20 and 20-30 cm at low-, mid- and high-altitude, respectively. Different lowercase letters represent significant differences with $P < 0.05$.
